# ‘Making time’: Long-distance marriages and the temporalities of the transnational family

**DOI:** 10.1177/0011392118792927

**Published:** 2018-09-13

**Authors:** Kristel Anne Acedera, Brenda SA Yeoh

**Affiliations:** National University of Singapore, Singapore

**Keywords:** Communication technologies, intimacy, long-distance marriages, temporalities, transnational family, Temporalités, mariages à distance, famille transnationale, technologies de communication, intimité

## Abstract

By focusing on the relations of intimacy between migrant wives working in Singapore and their left-behind husbands living in the Philippines, this article investigates how transnational couples negotiate the liminalities and temporariness embedded in the experience of labour migration. Using the timescales of migration as a conceptual frame, the article analyses the mutual, if uneven, shaping of marital relationships at the micro-timescale of transnational family time and the meso-timescale of Singapore’s labour migration regime. It focuses on how ‘doing family’ across distance is centrally facilitated through the affordances of communication technologies to create rhythms and manage ruptures. These technologies are crucial in (re)making domestic family time in the transnational household. The way the micro-temporalities of transnational family life are reorganised works in tension with how couples negotiate liminal conditions imposed by Singapore’s work permit and pass system. The article argues that temporariness and precarity, which deter the imagination of a stable future, are constantly negotiated in the lives of the transnational family through different temporal strategies. By bracketing off intense emotions and downplaying ruptures in relationships, the transnational family is able to focus on their future aspirations of achieving their projects through migration. As migration timelines are indefinitely extended and family separation is prolonged, the transnational family strives to endure through these strategies of (re)making their temporalities.

## Résumé

A travers les relations intimes entre les femmes migrantes qui travaillent à Singapour et les maris qui sont restés aux Philippines, nous avons étudié comment les couples transnationaux négocient les liminalités et la temporalité dans les expériences de migration de travail. En prenant les termes de la migration comme cadre conceptuel, ce travail analyse la conformation inégale des relations conjugales à la micro-échelle temporaire de la famille transnationale et à l’échelle moyenne du régime de travail des migrants à Singapour. Nous nous concentrons ainsi sur la manière dont les possibilités des technologies de communication permettent de créer des rythmes et de gérer les pauses pour « faire famille » à distance. Ces technologies sont cruciales dans une (re)construction du temps domestique dans le foyer transnational. La manière de réorganiser les micro-temporalités de la vie familiale transnationale fonctionne en tension avec le mode dont le couple négocie les conditions liminaires imposées par les permis de travail et le système des laissez-passer à Singapour. Nous soutenons que la temporalité et la précarité, qui dissuadent l’imagination d’un avenir stable, sont en permanence négociées dans « l’intimité stratégique » de la vie de la famille transnationale. En empêchant leurs émotions plus intenses et en minimisant les ruptures dans les relations, la famille transnationale parvient à se concentrer sur ses aspirations futures pour réaliser ses projets migratoires. Étant donné que les délais de migration s’étendent indéfiniment et que la séparation de la famille se prolonge, la famille transnationale s’efforce de soutenir ces stratégies de (re) construction temporaire.

## Resumen

A través de las relaciones íntimas entre las mujeres migrantes que trabajan en Singapur y los maridos que se quedaron en Filipinas, investigamos cómo negocian las parejas transnacionales las liminalidades y la temporalidad en las experiencias de migración laboral. Tomando los plazos de la migración como marco conceptual, este trabajo analiza la desigual conformación de las relaciones maritales en la micro-escala temporal de la familia transnacional y en la meso-escala de del régimen laboral de los migrantes en Singapur. Nos centramos así en cómo las posibilidades de las tecnologías de la comunicación permiten crear ritmos y administrar rupturas para “hacer familia” a distancia. Estas tecnologías son cruciales en una (re)construcción del tiempo doméstico en el hogar transnacional. La forma de reorganizar las micro-temporalidades de la vida familiar transnacional funciona en tensión con el modo como la pareja negocia las condiciones liminales impuestas por los permisos de trabajo y el sistema de pases en Singapur. Argumentamos que la temporalidad y la precariedad, que disuaden la imaginación de un futuro estable, se negocian permanentemente en la ‘intimidad estratégica’ de la vida de la familia transnacional. Inhibiendo sus emociones más intensas y restando importancia a las rupturas en las relaciones, la familia transnacional logra concentrarse en sus aspiraciones futuras de cumplir sus proyectos migratorios. Dado que las líneas temporales de la migración se extienden indefinidamente y la separación de la familia se prolonga, la familia transnacional se esfuerza en soportar estas estrategias de (re)construcción temporal.

## Introduction

There is a chronic sense of moral panic that globalisation has eroded traditional forms of kinship and familyhood, leaving no room for authentic intimate relationships to develop. However, rather than undermining intimacy, many scholars argue that globalisation is in fact merely reconstituting forms of ‘doing’ intimacy and familyhood (Constable, 2009; Walsh, 2009; Yeoh et al., 2005). The complex and unparalleled migration flows within and beyond Asia have given rise to ‘the geographically dispersed “family” as a “new” form of living arrangement’ (Yeoh, 2009: 1). This illustrates what Massey (1994: 4) describes as the ‘spatial reorganization of social relations’. The transnational family, as a social formation that derives its lived reality not only from material bonds of collective welfare among physically dispersed members but also a shared imaginary of belonging, features among both professional elite and low-waged migrant worker families.

In this article, we not only examine the reorganisation of intimate relations, but also endeavour to highlight the power-laden role of communication technologies in shaping the temporalities of transnational family life. Using case studies of Filipino migrant women working in Singapore, who negotiate long-distance intimate relationships with husbands and partners in the Philippines, we investigate the various strategies employed to (re)create the temporalities of the transnational family. We examine how they simultaneously attempt to strike a precarious balance between ‘doing’ family and achieving their migration projects.

Amidst the burgeoning literature on transnational migration and familial relationships, the preoccupation with space – how people, relationships, objects and ideas transcend, cross and re-cross spatial borders – has been a constant refrain (May and Thrift, 2001). More recently, scholars have argued for the need to attend to the relationship between migration and temporalities, given that migration is also a ‘process as much concerned with time as it is with space’ (Roberts, 1995: 42). Thus far, much less has been written about how the transnationalising family contends with the temporalities of migration. Following scholars like Cwerner (2001) and Robertson (2014a), this study aims to contribute to the agenda of forwarding migration studies beyond a preoccupation with space by asking ‘how do temporalities shape migration?’

Here, if we are to use temporalities as a heuristic tool for analysis, we must first clearly define what we mean by time and temporalities in this article. The difficulty in understanding how time shapes our everyday lives stems from how it elides analysis and problematising because it is often regarded as a ‘natural’ background of our everyday lives. A naturalistic view arises from a positivist tradition of time that is homogeneous, uniform and absolute because it is objectively and scientifically measured according to the movements of the sun and the cycle of seasons. In contradiction to an understanding of time as a naturally occurring backdrop to the unfolding of everyday life, we join other scholars in proposing instead that time is a tool for social control, creating multiplicity and unevenness in the political, gendered and racialised experience of temporalities (Lyman and Scott, 1989; Zerubavel, 1979). As such, in this article, we will deploy the term ‘temporalities’ to refer to how ‘time is neither objectively given as “real” time nor subjectively inaccessible as “lived” time, but rather is constructed as a cultural object and internalized as part of self’ (Rutz, 1992: 3). By underscoring the social construction of time through the term ‘temporalities’, we aim to problematise the temporal structures and boundaries used as tools to discipline social units such as the family.

In this article, we explicitly explore how temporal structures and boundaries of migration shape the intimacies of the Filipino transnational family. Not only are the spatial aspects of the family altered when members are dispersed across different nations, the temporal dynamics of the family are also reconstituted, as members rework and reimagine their familial relationships across different temporalities, negotiating the rhythms and tempo of everyday family life from afar.

In exploring the reorganisation of domestic and intimate lives in globalising households, scholars have noted that the role of communication technologies is becoming more pervasive. The plethora of available social media applications and communication technologies has made long-distance communication immediate and simultaneous. Technologies have acquired a form of ‘taken for grantedness’ (Ling, 2012), becoming cheaper, smaller and more mobile. In turn, long-distance communication has become a normalised and ambient feature of migration and transnational relationships. At the same time, these technologies are not simply neutral channels for communication, but are embedded in power geometries and often act as ‘tools of power’ and ‘vehicles of inequalities’ (Ducey, 2010: 22).

We use the experience of Filipino labour migrants in Singapore in ‘doing’ family vis-a-vis family members at home to understand how the temporalities of migration are shaping the transnational family. As one of Asia’s largest migrant-sending countries, the Philippines is lauded as a pioneer in the development of a highly institutionalised and regulated international migration framework that is geared towards the efficient deployment and protection of labour migrants (Asis, 2008; Battistella and Paganoni, 1992; Martin et al., 2004). Labour migration in the Philippines has had a long history of being deployed as a stop-gap measure to solve economic problems, such as the high unemployment and poverty rate in the country (Tyner, 1996). Reflecting the over-dependence of the Philippine economy on exporting labour, the Philippine Overseas Employment Association (POEA) reported that in 2014 alone, the agency processed and legally deployed 1.8 million overseas Filipino migrant workers. The top three occupations were domestic workers, nurses and food-service workers – all of which were notably feminised industries (POEA, 2014).

In ways which parallel the institutionalisation of overseas labour migration in the Philippines, Singapore as a receiving city-state has created a calibrated labour migration scheme to compete effectively in the global labour market in line with its aspirations to become a world city. Given Singapore’s strong demand for foreign labour – whether in domestic, service or professional work – it is unsurprising that the city-state was ranked among the top three destination countries for land-based Filipino migrant workers, with 125,320 Filipinos deployed to Singapore in 2014 (POEA, 2014). A differentiated system of employment passes and work permits regulates Singapore’s labour migrants according to a fine-grained continuum of (un)desirability. On one end of the spectrum, professional and highly skilled workers (considered ‘desirable’ bodies valorised for their talent) are governed by policies that allow for job mobility, visas for dependants and pathways to permanent residency. At the other end, low-skilled migrant workers are ‘needed’ but not ‘wanted’, and prevented from incorporation into the host country by policies that produce transience, such as short-term permits, workplace quotas and levies, and that close off the options of permanent residency or bringing in family members as dependants.

Singapore’s issuance of work passes and permits for foreign labour migrants is skills-based and salary-based. The Employment Pass is granted to foreign professionals (mostly in managerial, supervisorial, or executive positions) earning a salary of at least S$3300 a month. The S-Pass is granted to foreign mid-level skilled staff (in technical positions) with a salary of at least S$2200 a month. Meanwhile, the work permit category is divided into two: (1) foreign workers (specifically in construction, manufacturing, marine, process or service workers) and (2) domestic workers. There are no qualifying salaries required for either of these categories of work permits. However, in the case of Filipino domestic workers, the Filipino government has mandated a minimum monthly salary of S$550 for its nationals.

The type of pass and permit a foreign labour migrant has determines his/her eligibility to settle as a permanent resident in Singapore and to apply for Dependant’s Pass or Long Term Visit Pass (LTVP) for their family members. Employment and S-Pass holders are eligible to apply for Permanent Residence status, an option not available for Work Permit holders. Meanwhile, Dependant’s Pass (for spouses and children) or LTVP (for parents, common law partners and step-children) can only be applied for by Employment and S-Pass holders (with a monthly income of at least S$5000). Not only does this calibrated visa scheme structurally shape migrants’ work and living experience in Singapore, we argue that it significantly alters the way the transnational family conceptualises and experiences temporal rhythms and ruptures.

As discussed in the article, both the institutionalisation of labour export in the Philippines and the calibrated regulation of the import of labour in Singapore intersect in shaping the temporalities of the transnational family. In particular, we examine how migrants and their family members are embedded in and disembedded from different temporalities – of homeland and host society, and of the private and public spheres – and give attention to the ways migrant wives and left-behind husbands attempt to resynchronise disrupted temporalities through the use of communication technologies. In the next section, we turn first to examine the literature on temporalities, communication technologies and the transnational family.

## The temporalities of the transnational family

The temporal components of the visa and passes system constrain migrants under Employment Pass, S-Pass and work permits according to different levels of (un)desirability of the migrant body. This system produces heterogeneity and multiplicity in the temporal experiences coexisting in the same society and space. As Lyman and Scott (1989: 212) argue, ‘moments of time are conceived of as qualitatively uneven’. Ruled by visa and immigration policies, temporalities become central tools of discipline (Cwerner, 2001) that underline the migrant experience, ‘construct[ing] and constrain[ing] migrant biographies and trajectories of belonging’ (Robertson, 2014b: 1929). This inevitably shapes the meaning and practice of familyhood. By looking into how communication technologies are deployed to negotiate with the temporalities of transnational intimate lives, we examine how long-distance couples navigate the temporal dimensions of intimacy by deploying different synchronous and asynchronous affordances of communication technologies.

Migration – as an act of uprooting and regrounding not only in the spatial but also in the temporal context – is a process ‘where much of social life is potentially disrupted’ (Cwerner, 2001: 15). As migrants experience a disruption of the temporal context, the temporal orderings of both home and host societies also undergo some degree of flux. This results in what Gasparini (1994: 420, as cited in Cwerner, 2001: 8) describes as a ‘coexistence of different temporal orientations and actions that live together at the heart of a complex temporal architecture’. The superimposition of different temporal grids on the life worlds of migrants and their families often leads to disruption and fragmentation in experiencing the past, present and future. This requires individuals to negotiate with different temporal rhythms to create temporalities that make the most sense to their own life projects.

To provide a conceptual frame to analyse the temporalities of transnational familyhood, we draw from Robertson’s (2014a) work on the ‘Temporalities of international migration’ and Cwerner’s (2001) ‘The times of migration’. Robertson (2014a) employs Meeus’s (2012) concept of the ‘timescale’, which argues that the temporal organisation of migration happens at three levels: macro-timescale (global political scale), meso-timescale (national and supranational governance systems) and micro-timescale (community scale). Based on the idea that the temporal is a tool of discipline, Robertson (2014a) argues that the temporal orderings at the macro-level consequently influence the meso-timescales, inevitably shaping the minute and mundane orderings of the micro-timescale of family lives. The scope of this article will focus on the dynamics between the meso-timescale of the Singaporean immigration regime and the micro-timescale of transnational familyhood.^1^

As much as this study is about temporalities, it is also about communication technologies. Recent literature on communication technologies and intimate communication points us to an intensifying form of ‘time–space compression’. Newer, cheaper and more advanced forms of communication technologies – from telephones and smartphones to tablets, voice and video calls to social networking sites – afford ever-widening opportunities to keep in touch. The availability of a plethora of communication technologies gives rise to what Madianou and Miller (2013) term the integrated media environment of the ‘polymedia’. That there are multitude ways to maintain ‘near-perpetual contact’ (Katz, 2011: 306) and an ‘always on’ presence through communication technologies (Madianou, 2014: 674) shifts the focus of transnational communication away from questions of access and moves it towards *how* and *why* people choose the media they use. The ability to actively fashion one’s polymedia environment brings to the fore how we become ‘morally responsible’ for how, when and why we communicate (Madianou and Miller, 2013; Miller et al., 2016; Donath and Boyd, 2004). As the way we choose and layer communication technologies not only influences how we communicate, but more importantly shapes the intimate relationships we foster across time and space, we attempt to analyse how transnational couples reorganise their mediated intimate relationships. Communication technologies are not just neutral tools but vehicles of power. The way these technologies’ emotional, social and temporal affordances are harnessed provides insight into the underlying power geometries that shape the transnational family.

In considering the temporal affordances of communication technologies, Massey (1994: 149) reminds us to guard against the naïve celebration of a technologically driven universal ‘time–space compression’, and be more sensitive instead to the ‘power geometries’ that result in a ‘differentiated mobility’ in the experience of speed and distance. She argues that different individuals and organisations are placed in different positions relative to the flow of power to control and mobilise the compression of time and space through technologies. In her words, ‘some are more in charge of it than others; some initiate flows and movement, others don’t; some are more on the receiving end of it than others; some are effectively imprisoned by it’ (Massey, 1994: 149). In more specific terms, we explore the power geometries at play in negotiating the temporal capacities of communication technologies used in sustaining transnational families.

Drawing on the notion of multiple timescales as a broader context, we foreground the following concepts from Robertson (2014a) and Cwerner (2001) as an aid to capturing the temporalities of transnational familyhood, namely: rhythms, ruptures, liminality and simultaneity. We aim to understand how these simultaneously, if unevenly, influence and are influenced by how the transnational families shape their polymedia environment. Since this study focuses on the reorganisation of intimate lives in the domestic household across transnational space and time, we aim to unpack first *how* the micro-temporalities of familyhood are lived and structured in the banality of everyday transnational lives. We use the concept of ‘rhythms’ to capture the tempo and cadence of how everyday familyhood is lived, through maintaining serialised habits and routines that are repeatedly and cyclically done throughout a day or week. Conversely, we use ‘rupture’ to refer to the moments when rhythms become out-of-sync due to serious (e.g. relationship-ending) conflicts or more minor misunderstandings, which may create gaps or increase distance in transnational marital and familial relationships.

By paying attention to the way the micro-timescale of the everyday lives of transnational couples is predicated on the meso-timescale of immigration policies in Singapore, we highlight what Cwerner (2001: 24) refers to as the consistent challenge for migrants and their families to ‘transform the grey sense of meanwhileness into a vivid sense of simultaneity’. We explore the idea of migrants’ temporal negotiation between simultaneity and liminality in enabling transnational couples to imagine futures for themselves and their families. We argue here that the everyday banal temporal orderings of transnational familyhood using communication technologies lead to both rhythms and ruptures in the migrant’s experience of time, which in the end creates feelings of simultaneity or liminality.

Here, we want to highlight that achieving synchronous communication (the technological affordance of achieving immediate and simultaneous communication through voice or video calls) cannot simply be equated to achieving ‘simultaneity’. Cwerner (2001) refers to the sense of simultaneity as how migrants and their left-behind family members can continue to negotiate a life where their aspirations of migration coexist with the goals of the family. This creates a ‘sense that others are doing at the same time things that are meaningfully related to your own experience’ (Boyarin, cited in Cwerner, 2001: 23). By creating a sense of simultaneity, migrants and their families feel that they are not trapped in a spatio-temporal liminality, where they are isolated not only from one another but from the host society as well. In short, transnationally separated family members can have synchronous communication, yet not feel a sense of simultaneity with one another in terms of their imagination of the present and the future. By fashioning a sense of simultaneity in everyday rhythms through communication technologies, members of the transnational family are able to better negotiate their migration and family life trajectories. In contrast, liminality pertains to how the temporariness that is embedded in the meso-timescales of Singapore migration policies disables the transnational family from living a life where family migration projects can be fulfilled. Cwerner (2001: 27) defines liminal times as the transitional condition, especially in temporary migration, where migrants and their left-behind family members are ‘always “making up their minds” ’, stuck in moments of ‘indecisions, confusion, incompleteness, underachievement and eternal expectation’. Liminality creates moments when the migrants have to decide whether to suspend or abandon migration projects in order to sustain family togetherness, or allow the family to break apart while pursuing individual pathways opened up by migration possibilities.

## Methods

This study is based primarily on qualitative data gathered from November 2014 to July 2015 across two sites: interviews in Singapore with migrant wives and interviews in the Philippines with left-behind husbands. Purposive sampling was employed to recruit participants in order to be able to deliberately train our lenses on the impact of the temporal aspects of migration on long-distance intimate relationships. This study relied on key informants and the snowballing technique to locate and access participants. The following were the sampling criteria of the study:

1. To examine feminised labour migration in Singapore and its impact on left-behind families in the Philippines, this study was limited to only Singapore-based migrant women and Philippine-based left-behind men.
2. The participants in this study had an experience of long-distance marriages that ranged from at least 2 to 15 years.
3. The study was limited to participants who had been married or cohabiting (for a minimum of 3 years).

In total, we conducted 30 qualitative life story interviews. Of this sample, 15 were migrant wives and 15 were left-behind husbands. Five couples were paired, while the remaining were unpaired and unrelated. Tables 1–3 present the profiles of the participants.

**Table 1.** Profiles of the paired transnational couples.

	Name	Age	Occupation	Visa type	Name	Age	Occupation	Children	Status
1	Lovely	44	Domestic worker	Work permit	Gerardo	43	Farmer	4	Cohabiting
2	Joanne	32	Front desk officer	S-Pass	Carlo	41	HR manager	1	Cohabiting
3	Sheila	31	Marketing officer	Employment Pass	Robert	40	Church volunteer	0	Married
4	Janna	28	Domestic worker	Work permit	Andrew	30	Garbage collector	3	Married
5	Analyn	35	Domestic worker	Work permit	Manuel	40	Farmer	3	Married

**Table 2.** Profiles of the unpaired migrant wives.

	Name	Age	Occupation	Visa type	Children	Status
1	Charlene	34	Casino supervisor	S-Pass	1	Married
2	Rowena	34	Domestic worker	Work permit	1	Married
3	Melanie	27	Front desk officer	S-Pass	0	Married
4	Graciel	41	Domestic worker	Work permit	4	Married
5	Bernice	30	Casino beverage staff	S-Pass	2	Cohabiting
6	Jessa	34	Medical technologist	Employment Pass	3	Cohabiting
7	Dina	26	Casino beverage staff	S-Pass	1	Cohabiting
8	Noreen	32	Domestic worker	Work permit	1	Married
9	Genevieve	34	Educator	Employment Pass	1	Married
10	Donna	40	IT manager	Employment Pass	1	Married

**Table 3.** Profiles of the unpaired left-behind husbands.

	Name	Age	Occupation	Partner’s visa type	Children	Status
1	Daniel	36	Entrepreneur	Employment Pass	3	Married
2	Ricardo	49	Unemployed	Work permit	4	Married
3	Randy	50	Unemployed	Work permit	3	Married
4	Joseph	26	Waiter	Work permit	1	Married
5	Gino	31	Unemployed	Work permit	2	Married
6	Jonathan	39	Farmer	Work permit	1	Married
7	Neil	40	Policeman	S-Pass	4	Married
8	Albert	36	Factory worker	Work permit	1	Cohabiting
9	Noel	34	Call centre agent	S-Pass	1	Cohabiting
10	Francis	45	Insurance collector	Work permit	2	Cohabiting

Among paired interviewees, having the perspective of both migrant wives and their left-behind husbands allowed us to gain insight into family dynamics and how migration simultaneously affects those who move and those who stay behind. Meanwhile, including unpaired couples allowed more room for freedom and candour, as the participants’ fear of being exposed to their partners was lessened. To understand how different labour migration policies, visa conditions and social class affect the performance of gender identities in intimate relationships, the migrant participants were also drawn from different migration categories: Employment Pass (professional), S-Pass (mid-skilled technical staff) and work permit holders (low-skilled). The (un)employment of the left-behind men was left open to variance to examine what types of livelihoods men took on in the wake of their wives’ migration.

The main research tool for data collection was the semi-structured life story interview. In many ways, life story interviews projected the voice of the participants, as it allowed them to narrate stories in an organic way. The interview guide touched on the following themes: childhood family experiences; education and employment history prior to migration; love and married life stories; migration history; rituals and routines of transnational family; and communication technology use and practices. The interviews were conducted in a mix of Filipino and English. These were audio recorded, anonymised and then thematised. We paid attention to major themes that recurred across the interviews, but also took note of ‘outliers’ and unusual situations in the stories. The interviews lasted from one and a half hours to three hours, with some respondents being interviewed more than once.

The interviews with migrant women in Singapore and left-behind husbands in the Philippines were conducted by one of the authors, who is Filipino, single and in her twenties. Having lived in Singapore for several years, she had access to a range of participants from different backgrounds. At the same time, the interviewer’s positionality influenced the fostering of camaraderie and trust with participants. Her social class background – coming from an exclusive university in the Philippines and being ‘privileged’ to study in Singapore – made it tricky to navigate fieldwork especially with domestic workers. Although participants were hospitable and friendly since the interviewer was another Filipino, there was a power imbalance in how they either deferred to the interviewer as an ‘educated’ researcher or they became aloof, treating her as an outsider. This was overcome by building more trust and camaraderie through joining them in other activities outside the research setting. Meanwhile, being young, female and unmarried, the researcher was presented with some challenges in gaining access to the left-behind husband participants. On one level, there was some apprehension among the wives in introducing the researcher to their husbands, given how their long-distance relationship made the interviewer appear as a ‘temptation’ or potential ‘threat’ to their marriage. However, when access to the husbands was granted, the gender difference did not seem to affect the interviews., The men expressed a sense of relief in their willingness to discuss marital and household issues with a ‘stranger’, given the immense difficulties they encountered in sharing with friends and family due to the silencing effect of shame.

## Rhythms: On how communication technologies resynchronise family temporalities

In this section, we examine how the everyday micro-timescale of the family is reordered and negotiated in ‘doing family’ transnationally. Particularly, we analyse how the temporal boundaries and structures of migration (i.e. time zone difference, days off, visa and immigration policies) shape the intimate relationships in the transnational family. We contend that the way transnational couples prioritise *what, how* and *when* activities and schedules should be resynchronised in the transnational household reflects how intimate familial relationships are changing during the period of migration.

### On being ‘near’

Consistently, most of the participants were quick to explain that ‘earning and saving more money’ was the primary reason leading to the choice of a transnational family lifestyle. What was however more striking was the fact that in the case of both migrant wives and left-behind husbands, the second most commonly mentioned reason for migration to Singapore was, ‘It’s near’. When asked to share how migration was decided, both migrant wives and left-behind husbands pointed that Singapore had the key advantage of being just a three-hour flight away. A constant refrain was that spatial proximity allowed for the possibility that migrant wives could fly home should there be a sudden need for them to be physically back home. Given the rapid expansion of budget airlines in the region, it is relatively cheap and less time-consuming to travel between the two countries when compared to other top migrant destination countries in the Middle East, the United States and Europe. As the homeland is perceived to be nearby, Singapore’s distance from the Philippines fosters a sense of security in the minds of the transnational couple. Ricardo (49 years old, unemployed) explained:

You keep on hearing about abuses happening [to migrants] in Saudi Arabia and other countries. … I mean, in Singapore that can still happen. But at least she’s just near. The fact she can come home easily or maybe I can even fly in if she needs me … that gives me some peace of mind.

In a way, this meant that help was perceived to be more easily available due to proximity, in case of abuse, mistreatment, or other unfortunate events that might happen during migration.

The case of Donna (38 years old, IT manager, Employment Pass) is illustrative of how migrant women rationalised Singapore’s ‘nearness’. When Donna’s husband became bed-ridden due to an illness, she made sure that she was in the Philippines once or even twice a month, usually during long weekends, to check that her husband was being given proper care. This is despite having already employed a full-time caregiver for her ailing husband. Due to her managerial position, Donna can be considered a ‘mobile’ migrant. Her salary, leave allocations and ability to schedule her vacations whenever she wanted to have allowed her to take advantage of Singapore’s proximity to the Philippines. For Donna, the value of being able to ‘be there’ physically for her family was an important deciding factor in favour of Singapore than the potential of getting a higher salary elsewhere. She explained:

I’ve been getting a lot of job offers to work in Canada, the USA, even in Europe! I do think about how big these opportunities are. Really high salaries! Better benefits! But you know it’s just too far … half-way across the world! Had I been younger and single, I would have grabbed these offers in a heartbeat. But now, I have a husband and a son back home to think of …

However, we argue here that in many instances, what is important is not the *actuality* of coming home, but the *potential* of being able to come home. The home – as a physical place and a symbol of family life – was seen by migrant women to be crucial to their identities as mothers and wives. This resonates with the idea that the domestic sphere is socialised to become the ‘naturalised’ sphere of mothers and wives; while the public sphere of work is deemed to be the domain of men (Bourdieu, 2001). When wives migrated not only out of the homeland but out of the home, which was associated with their primary identity as homemakers, they often experienced a sense of guilt and ambivalence. The importance of the *potential* to come home, then, needs to be understood in the context of migrant wives’ attempts to find a middle ground between their new identities as primary breadwinners, while tempering the guilt of not being able to be present in the homespace.

In reality, despite the spatial proximity of Singapore to the Philippines, returning home on a regular basis was out of the question for most of the domestic worker participants, as they were both time-strapped and money-scarce. Given average incomes amounting to no more than S$550–750 a month, buying a return flight back home each month would not be practical. Aside from a once-a-week day off from work, domestic workers do not have annual leave allocations that will enable them to fly back home. They are also bound by a two-year contract in which they are allowed to fly back home only at the end of each contract. Because of this, domestic workers like Rowena (34 years old, work permit) feel the need to plan *when* exactly they would be home to make the most of their limited time. This becomes an issue of prioritising some family events over others, and becomes even trickier when sudden family emergencies arise. Rowena’s unexpected emergency came when she heard reports of her husband allegedly bringing another woman to live with him in their house. Rowena was in distress, but when asked whether she would go home to deal with this issue, she rationalised:

No, I don’t think I can come home right now. My employers and her children have been very concerned. They said, ‘Auntie, go home. Try to fix your marriage yourself.’ They see that I’m very sad and I cannot sleep. But no, I don’t think I have enough money to fly home right now. Also, I have already promised my son that I will be home for his graduation … so maybe our marriage will just have to wait until then.

In this case, Rowena had to choose between flying home that moment or waiting to activate her original plan to go home for an important occasion.She made this decision being fully aware that her son’s graduation was still one year away. In a follow-up interview, Rowena shared that when her marital problems reached extreme heights, she could no longer afford to wait for her son’s graduation. She went home, and happily reported that her husband and herself have managed to save their marriage. She was faced with a choice. By going back home to fix her marriage, she had to give up her chance to witness her son’s graduation.

Even for participants working in the service industry who were able to command better salaries (from S$2000 and above), the actuality of going home more frequently was hindered by the limited number of annual leave days, which were also subject to their employer’s approval. The policies on leave per company vary, but a baseline number of seven days of annual paid leave is mandated by the Ministry of Manpower for the first year of service (MOM, 2015). While middling professional workers had more opportunities to return home compared to foreign domestic workers, having to seek the approval of employers even for a few days proved to be an obstacle at times. For example, participants who worked in the hotel and medical fields encountered difficulties in securing their employers’ approval for important festive seasons like Christmas and New Year when work demands tended to peak. In such cases, the lives of the migrant wives had to be put on hold. They were subjected to the temporal rhythms of their work to ascertain *when* it would be more convenient for their employers to accommodate their request for holiday leave. For many participants, it was during these times – when migrant wives and their family members ‘missed’ events that would allow them to celebrate familyhood together – that they felt entrapped in the ‘liminal times’ of migration.

Here we can see ‘power geometry’ (Massey, 1994) at work, as different temporal boundaries create differing ‘potential’ to come home for migrants in each visa category. Employment Pass holders have the ‘most potential’ to come home, while work permit holders have the ‘least potential’. Despite the Philippines being a three-hour flight away and even with the abundance of cheaper airfare flights, one’s mobility and potential to be near become relative to one’s social class position. As an IT professional carrying an Employment Pass, Donna’s case demonstrated how her class positioning (shown in her ability to afford plane tickets for weekend trips) and work benefits (her leave entitlements and salary) had allowed her to fully take advantage of the ‘potential for nearness’. She could come home and be physically present to take care of her ailing husband. As the minimum basic salary requirement for an Employment Pass is S$3600, they have a higher buying capacity to shoulder airfares and other transportation costs compared to S-Pass holders, who earn below this range. This allows them to be more mobile than S-Pass holders. In the case of domestic workers on the work permit scheme, their ability to afford plane tickets and the lack of annual vacation leave benefits strongly hampers the potential for nearness. Compared to Donna, Rowena’s decision to save her marriage is made complicated by temporal and mobility restrictions, forcing her to put intimate relationships on hold.

The meso-timescale of Singapore’s labour migration regime governs migrant bodies differently, making the experience of ‘nearness’ relative to one’s position in the labour market. Here, it is clear that ‘nearness’ becomes less about actual kilometres between two countries, and more about the relative degrees of freedom the migrants possess in managing how and when the spatial and temporal distance between home and host countries can be overcome. Massey’s (1994) concept of ‘power geometry’ is at work here: while some of our participants with more bargaining power at work have the ability to conquer the unevenness of space and initiate flows and movement, others are implicated in an unequal power hierarchy and rendered immobile.

### Negotiating the rhythms of temporal ‘mobile lives’

In this section, we analyse how migrant families negotiate the temporalities of migration (i.e. temporal boundaries and structures that shape the migrant experience) by deploying different (a)synchronous and affective affordances of polymedia. We argue that because of the differences in accessing ‘potential for nearness’, fostering the ‘imaginary of nearness’ through the use of communication technologies becomes all the more important for the migrant families. As with other empirical studies (Baldassar, 2008; Chib et al., 2014; Peng and Wong, 2013; Uy-Tioco, 2007; Wilding, 2006) that focus on communication within the transnational family, long-distance communication was found to be the constant and primary medium through which transnational relationships were conducted.

One key feature that largely shaped how the micro-temporalities of long-distance communication were reorganised in the transnational family life of Singapore-based migrants was the lack of time-difference between the two countries. Because of their spatial proximity, Singapore and the Philippines operate within the same zone of +8 Greenwich Mean Time (GMT). Couples do not have to keep track of dual time zones and adjust and negotiate a mid-way time that would be convenient for both parties.

To resynchronise their intimate lives and maintain ‘connected presence’ (Licoppe, 2004), both migrant wives and left-behind husbands shared how they used different technologies to fulfil different communication needs, depending on the urgency of the concerns to be managed and the time of communication. Usually, synchronous communication like calls (whether through free voice-enabled Skype or Viber, or through prepaid phone calls) were used for more leisurely conversations, often during days off and at the end of migrant wives’ work shifts. Meanwhile, asynchronous communication like leaving text messages on messaging applications such as Viber, Whatsapp and Facebook messenger was preferred by the left-behind men, as it allowed them to make their presence felt without having to disrupt the work flow of their wives.

Left-behind husbands whose wives were now the major breadwinners often tried to ensure that their wives’ work schedules were not disturbed. At the same time, they emphasised the need to assure their wives daily that they were always thinking of them. Robert (40 years old, church volunteer) shared how important it was to send simple messages such as ‘good night’ and ‘good morning’ each day. Robert insisted that without these ‘good morning’ and ‘good night’ messages, his wife would ‘throw a tantrum the whole day’, as the lack of the familiar messages was a signal that there was a break in their resynchronised lives. This kind of asynchronous communication through instant messaging or text messages was preferred, as it allowed transnational couples to communicate without disrupting their respective routines, schedules and activities.

Transnational couples also supplemented the bite-sized communication of messaging done throughout the day with more synchronous forms of communication through calls, either through voice-over IP applications like Viber, Skype and Facebook calls or through the traditional mobile phone calls using prepaid cards. Robert shared how as a long-distance couple, they have managed to build a communication routine throughout his wife’s migration stint:

So in the morning, with no fail, I will already give her a call on Viber to wake her up. Like an alarm clock everyday. I say good morning … then we start the day with a bible reading and our morning prayers. We do it together [on the phone]. We’ll talk until she finishes her breakfast and I finish mine. Then when she’s at work, we just send each other text updates … random stuff like what she’s eating for lunch or I tell her stories about her mother or the church group. Then at night, we call each other again either through Facebook or Skype. Just tell[ing] each other about our day. In the evenings, we have time to see each other through video calls. And then we do our bible sharing again. It’s very important for us. Then [we] sleep, then [we] start again.

It was interesting to note that in most cases, video calls through Skype were not a preferred means of communication either for migrant wives or left-behind husbands. Participants said that video calls took too much time to set up and required a specific coordination of time when transnational couples could sit down and be free from other distractions. Hence, calls without video were much preferred for daily communication, especially through free calls provided by Viber and Facebook. Melanie (27 years old, front desk officer) explained the logic behind preferring just audio-centric calls instead of the audio-visual affordance of communication technologies:

I actually like to just use Viber … I can just put on my headphones and multi-task like do my laundry, go to the grocery, cook. … I don’t want to waste my day-off just locked inside the house basically tied to my computer.

These findings on how the use of communication technologies and the choice of communication medium affected the temporal reordering of transnational intimate lives reflect Green’s (2002) research on how mobile technologies, specifically mobile phones, create what she calls ‘mobile time’. She argues that as communication technologies become smaller and more personalised, it has caused the blurring of private and public lives. This results in mobile time, whereby one is almost always accessible through mobile device/s, regardless of when or where. She hazards that this leads to work time expanding even as it becomes more flexible, as it encroaches on and even colonises private home life. In the case of migrant wives and left-behind husbands, the blurring of their private and public spatio-temporalities opened up opportunities for creating a shared temporal rhythm within the transnational family from afar, if in a strategic and managed manner.

The accounts shown above point us to how transnational couples strategically manage not only their temporalities, but also the kind of integrated polymedia environment they use to shape intimacy in transnational communication. This reminds us of what Madianou (2016: 183) calls an ‘ambient co-presence’ or the ‘increased awareness of the everyday lives and activities of significant others through the background presence of ubiquitous media environments’. No longer focused on only one kind of communication technology, the affective affordances of different communication technologies (whether audio, visual, textual, synchronous or asynchronous) are harnessed to create the kind of polymedia environment one sees fit to shape different forms of intimacies and transnational lives.

Delving deeper into how transnational couples reorganised intimate transnational temporalities, we see that the way they shaped their polymedia use is governed by the rhythms of their reconstituted transnational domestic lives in Singapore and the Philippines. One of the changes that influenced the micro-temporalities of transnational couples was that the migrant wives were now employed in occupations that had more rigid work hours, which limited communication time and frequency. Meanwhile, left-behind husbands usually changed to more informal means of work to accommodate the wives’ absence from home. Because of the strict work hours that migrant wives had to keep, as well as the notion that their work was now more valuable as the primary source of the family’s income, the migrant wives’ schedule inevitably dictated the rhythm and frequency of communication.

This is tied to the gendered value attached to work, where productive and economic work is seen as more valuable, and thus is given temporal priority, compared to reproductive and caregiving work that can be halted and disturbed to accommodate other more economically driven imperatives. In this case of role reversal, the prevailing gendered value of work that privileges productive over reproductive work meant that among most of the participants, migrant wives were the ones to dictate the time and frequency of transnational communication. Gino (31 years old, left-behind father), who quit his job in sales to take care of his children, explained:

I quit because someone had to take care of the kids. She was earning more as a domestic worker than what I could ever earn in my sales job. So it was better that it was me who quit. Since I’m just at home, for communication, we just follow her time there … whenever she’s free … whenever the boss is not there.

In sum, we argue that migrant women and left-behind men continue to create synchronous lives that foster the ‘imaginary of nearness’ through the use of communication technologies. Although the content of the communication shown here is more functional and mundane, by focusing on and analysing the rituals of the *how* and *when* of transnational communication, we gain insights into the values that are being prioritised and the status quo being (re)created in the transnational couples’ relationships.

## Ruptures: On the limits of communication technologies in keeping the family together

We turn in this section to examine the limits and pitfalls of communication technologies in resynchronising distanciated lives in moments when relationships are ruptured. In particular, we argue here how ruptures, conflicts and misunderstandings disrupt the synching of temporalities and intimate lives, preventing transnational couples from creating simultaneity. It is during moments of rupture when even the affordances of communication technologies fail to create an ‘imaginary of nearness’. Instead it makes more evident the spatial and temporal gaps between transnational couples.

### Mediated absence

Although Singapore and the Philippines are geographically near each other, the limited opportunities for some migrants to actually come home because of the temporal constraints of their jobs created room for feelings of loss, jealousy and mistrust to develop. In the instances when issues in the transnational relationship became thorny, long-distance virtual communication was inadequate to alleviate problems and hurt feelings that were usually resolvable only by face-to-face communication and physical intimacy.

With the availability of cheaper and more advanced mobile phones, tablets and computers, as well as the plethora of free messaging and social media applications, transnational couples affirmed that communication had become easier and instantaneous. However, similar to the studies of Horst (2006) and Tazanu (2015), communication technologies were still seen in an ambivalent light, as it confronted migrants with the kind of communication modes that were simultaneously ‘blessings and burdens’ (Horst, 2006). One key finding in this study was that the availability of cheaper and more advanced communication technologies, and the access to these, did not necessarily translate into more frequent communication that deepened bonds between transnational couples.

Indeed, while accessibility and affordability did open up ways to communicate with transnational partners, these did not always translate into actual communication, especially during moments of conflicts. During these times of tensions, the affordance of communication technologies to offer what Couldry (2004) referred to as ‘liveness’ was often seen by participants as a burden. This was because the simultaneous transmission and reception of realities into one another’s lives demanded immediate confrontation of problems that were not easily solvable when the transnational couples were apart.

While talking to Donna about who frequently initiated regular communication between her and her left-behind husband, she admitted that she initiated most of the conversations. She reflected on this:

Maybe he doesn’t initiate conversations anymore because I just end up nagging him on the phone about the things he did or did not do regarding the apartment or other household concerns that we always fight about. But if I don’t nag him, nothing gets done so I really have no choice.

With the reversal of breadwinning and caregiving roles between migrant wives and left-behind husbands, budgeting and finances become sensitive topics especially for left-behind husbands. As Gerardo (a 43-year-old farmer) shared:

My wife would always tell me that I should just stay at home and stop farming. I always get angry when she says that. … Yes, I don’t earn much, but let me keep my dignity [I don’t want to] feel like my balls have been cut off.

The form of resistance to the ‘*liveness*’ of communication was through the obvious display of *absence*. Instead of opening up communication lines during conflict, most participants disclosed that they tended to deploy strategies of making their dissatisfaction felt through what we call ‘mediated absence’. By this we refer to how they choose not to be accessible despite the plethora of transnational communication means that can connect them instantaneously. As discussed in the previous section, the option to layer different communication technologies to create an integrative polymedia environment creates an ‘ambient co-presence’ that makes it harder to be disconnected than connected. Hence, the deliberate avoidance and absence in communication, in itself, is used as a way to flag issues in the relationship. This was the case when couples harboured misunderstandings or were engaged in fights. Contrary to the hypothesis that the availability of communication might open channels for discussion of problems that cropped up in the maintenance of transnational relationships, the findings showed that both migrants and left-behind partners chose not to confront these problems by avoiding talking about them. This created asynchronous lives that made the temporal and spatial distance so much more evident.

The promise of constant and frequent connection brought about by the possibilities to communicate often heightened expectations that in turn led to disappointment and resentment between couples. Manuel (40 years old, farmer) shared his agony that he was confronted by a wall of silence despite the many options to communicate with his migrant wife:

To be honest, I’ve been losing sleep. … My wife ignores me and does not talk to me; I do not know what the problem is. I try to communicate with her. I have a lot of instant messengers, Viber, Tango, Facebook Messenger, Skype, name it I have it. I message her [in all her accounts] all the time. But she rarely replies to me. I know she sees it. I can see that she’s online.

Because communication could be easily had at virtually no cost at all, it made it hard for either party to understand the reason why their partners did not communicate with them. During a period of non-communication, trust was sometimes damaged as both parties began to imagine how the other was spending their time apart and suspicion set in. They wondered why their partners could not send even a simple message when there were so many ways to communicate.

Mediated absence can be easily created by not replying to text messages, not answering calls, or delaying any form of communication with transnational partners. Janna (a 28-year-old domestic worker), who was on the brink of ending her marriage with her husband Andrew, said that she chose to ignore and not reply to the many angry text messages she used to receive from him if only to prevent further arguments. She felt their issues could only be resolved by talking face-to-face when she returned home, and not through text messages and calls from afar. Meanwhile, when we interviewed her husband Andrew (a 30-year-old garbage collector), he cited Janna’s evasion as one of the reasons that he suspected she was having an affair in Singapore. Andrew shared, ‘Perhaps she is hiding something. … Or she’s with another man. No painful words get through to her anymore.’ Although there were indeed bigger marital issues that plagued the couple, the mediated absence deployed by Janna and her husband further intensified the anger and dissatisfaction that led to the dissolution of their union eventually. It seemed that attempts to resist one-way communication or ‘nagging’ via technologies more often than not created further conflicts instead of solving them.

Here lies the limit of communication technologies to bridge ruptures in relationships. At times, harnessing the temporal and affective affordances of communication technologies to create ambient co-presence is simply not enough to deal with marital issues that need to be dealt with face-to-face. Again, migrants in different categories are affected differently depending on how they can negotiate with the meso-timescale of Singapore’s migration regime.

This sense that the other becomes a ‘stranger’ is not only an emotional reaction, but is also embedded in the larger politics of Singapore’s migration policies. Those with Employment and S-Passes are in a position to negotiate with the meso-timescale of Singapore’s immigration regime, enabling them to harness the ‘potential for nearness’ not only spatially, but emotionally as well. Not only could these high- and mid-skilled migrants afford to go home, but they could also afford to fly their partners and families to Singapore for a vacation. This opened up opportunities not only to be reunited with their loved ones, but also on a deeper level, be reacquainted with each other by face-to-face experiences that can complement and augment their long-distance mediated relationships. In contrast, these options are not available to migrants under the work permit scheme, as they could neither bring their families to Singapore nor return home frequently to fix their marital problems.

Thus, we argue that as much as ‘connected presence’ is important in understanding how communication technologies bridge relationships, it is equally important to understand how ‘mediated absence’ presents a different perspective on the nature of intimacy in long-distance marital relationships within a changing spatial and temporal context.

Under Singapore’s labour-migration regime, based on a ‘use and discard’ policy (Yeoh, 2006), the calibrated visa and work permit scheme induces temporariness especially for those who are excluded from applying for Permanent Residence and LTVP for their family members, making it difficult to have a life together in Singapore. Separation due to migration is indefinitely extended – in the words of participants – in order to ‘seize the moment’. Here, participants referred to seizing opportunities that were deemed to be too good to pass up, like sudden extensions of contracts, job promotions and opportunities for employment. The moments to be ‘seized’ usually pertained to opportunities that were not originally part of the migration plan. But as these opportunities were often perceived to have considerable value in securing the family’s future, original plans of migration were readily altered.

Because of the lack of permanency in Singapore, when migrants were asked how long they saw themselves working overseas, their responses were usually based on the length of their work contracts. Longer term plans of five or ten years were often not considered, as migrants and their left-behind family members planned according to their one- to two-year contract period. The family’s future plans were often precariously pivoted on the temporary conditions of their work, and as such, both migrant wives and left-behind family members did not have the assurance of any degree of permanence in plans. Instead, plans were often spontaneous, ad hoc and flexible in nature. It was not unusual for left-behind family members to have to cope with many difficulties synchronising their goals according to the ‘original’ plan as this was always in the process of being revised. Transience and precariousness thus defined the temporality of the transnational family in liminal times.

## Conclusion

This article has examined the intersection between transnational families’ temporalities and the affordances of communication technologies. One key argument is that ‘doing family’ is centrally facilitated through the affordances of communication technologies to create rhythms and manage ruptures. The way the micro-temporalities of transnational family life are reorganised ultimately defines how couples negotiate liminal conditions imposed by the meso-timescale of Singapore’s temporary migration regime. First, in examining how transnational families resynchronise disparate temporalities across space, we show that the imaginary of ‘nearness’, and not the ‘actuality’ of being near their loved ones, is more important in assuring transnational family members of a sense of control over their own lives despite temporal and spatial distance. Second, while communication technologies have enormous potential to create synchronised lives, they are also complicit in the way conflicts, misunderstandings and ruptures are produced in transnational intimate relationships. In fact, communication and the expectations generated by advancements in technology may become the crippling factors during times of rupture. Third, we argue that the micro-temporalities of transnational families’ everyday lives cannot be understood without considering the meso-temporalities of immigration policies that instil temporariness and precarity in the lives of the transnational family, deterring the imagination of stable family futures.

By examining the temporal structures and boundaries that undergird the everyday lives of migrant families, we contribute to the literature on migration, new media studies and transnational families, in three ways. First, this article engages with the migration literature by foregrounding the interplay between agency and structure in how migrants and their families negotiate with the boundaries and borders of migration. By shifting the focus beyond space to include temporalities, we highlight how (im)mobilities are not only shaped by spatial boundaries, but also produced by temporal boundaries and structures. As such, we want to argue that a sense of (im)mobility is not only determined by one’s movement through space, but also movement through the temporal – by the degree of power to negotiate the speeds and pauses of one’s past, present and future temporalities. What we argue here is that the inherent temporariness in Singapore’s migration regime not only shapes the lives of migrants in the host country, but also significantly creates temporariness and precarity in the everyday lives of the family left in the homeland.

Second, this article draws on the burgeoning literature on communication technologies to argue that transnational communication is not simply about ‘time–space compression’. Rather, by looking at the temporal and affective affordances of communication technologies to create rhythms and manage ruptures in intimate relationships, we highlight how technologies are ‘time-structured’ and ‘time-structuring’ (Silverstone, 1993) tools that shape and are shaped by the power dynamics of the transnational family. We argue for a rethinking of communication technologies not only in terms of connectivity – being ‘always on’ and having ‘ambient co-presence’ – but also in terms of how mediated absence is a communication strategy that can be telling of the existing power dynamics in transnational communication.

Finally, by examining the micro- and meso-timescales of migration, we aimed to contribute to the literature on transnational families by highlighting the temporal aspects of the strategies involved in migration projects. The power geometry undergirding the labour migration regime shaped two migrant temporalities: that of the *present* (which related to migrants’ current everyday realities and lived experience) and the *future* (which related to their aspirations and future imaginaries) for migrants and their families. These two aspects of the micro-timescale of migration were directly affected by the meso-timescale of immigration policies in Singapore that instilled temporariness in the current everyday lives of migrants, and also created uncertainties in their imaginaries of their future. By examining how transnational families used communication technologies to manage marital relationships from afar, it became clear that how temporalities were synchronised or disrupted in migration played an important role in creating or fracturing intimate proximities. Sustaining the hope of familyhood in future temporalities was predicated on fostering family rhythms and modulating feelings of rupture, as a way of negotiating the temporal structures written into Singapore’s labour migration regime.

## Notes

1. Although we acknowledge that the global macro-timescale undergirds the meso- and micro-timescales of the transnational family, this article will specifically only focus on how the temporal boundaries of Singapore’s meso-level immigration and visa policies inevitably shape the intimate orderings of the micro-timescale of the family.
